# Periodontitis: etiology, conventional treatments, and emerging bacteriophage and predatory bacteria therapies

**DOI:** 10.3389/fmicb.2024.1469414

**Published:** 2024-09-26

**Authors:** Anna Łasica, Piotr Golec, Agnieszka Laskus, Magdalena Zalewska, Magdalena Gędaj, Magdalena Popowska

**Affiliations:** ^1^Department of Bacterial Genetics, Institute of Microbiology, Faculty of Biology, University of Warsaw, Warsaw, Poland; ^2^Department of Molecular Virology, Institute of Microbiology, Faculty of Biology, University of Warsaw, Warsaw, Poland; ^3^Triodent Dental Clinic, Warsaw, Poland; ^4^Department of Bacterial Physiology, Institute of Microbiology, Faculty of Biology, University of Warsaw, Warsaw, Poland

**Keywords:** antibiotic resistance, bacterial biofilm, bacteriophage therapy, inflammation bacteria, oral microbiome, periodontal diseases, predatory bacteria, risk factors

## Abstract

Inflammatory periodontal diseases associated with the accumulation of dental biofilm, such as gingivitis and periodontitis, are very common and pose clinical problems for clinicians and patients. Gingivitis is a mild form of gum disease and when treated quickly and properly is completely reversible. Periodontitis is an advanced and irreversible disease of the periodontium with periods of exacerbations, progressions and remission. Periodontitis is a chronic inflammatory condition that damages the tissues supporting the tooth in its socket, i.e., the gums, periodontal ligaments, root cementum and bone. Periodontal inflammation is most commonly triggered by bacteria present in excessive accumulations of dental plaque (biofilm) on tooth surfaces. This disease is driven by disproportionate host inflammatory immune responses induced by imbalance in the composition of oral bacteria and changes in their metabolic activities. This microbial dysbiosis favors the establishment of inflammatory conditions and ultimately results in the destruction of tooth-supporting tissues. Apart microbial shift and host inflammatory response, environmental factors and genetics are also important in etiology In addition to oral tissues destruction, periodontal diseases can also result in significant systemic complications. Conventional methods of periodontal disease treatment (improving oral hygiene, dental biofilm control, mechanical plaque removal, using local or systemic antimicrobial agents) are not fully effective. All this prompts the search for new methods of therapy. Advanced periodontitis with multiple abscesses is often treated with antibiotics, such as amoxicillin, tetracycline, doxycycline, minocycline, clindamycin, or combined therapy of amoxicillin with metronidazole. However, due to the growing problem of antibiotic resistance, treatment does not always achieve the desired therapeutic effect. This review summarizes pathogenesis, current approaches in treatment, limitations of therapy and the current state of research on the possibility of application of bacteriophages and predatory bacteria to combat bacteria responsible for periodontitis. We present the current landscape of potential applications for alternative therapies for periodontitis based on phages and bacteria, and highlight the gaps in existing knowledge that need to be addressed before clinical trials utilizing these therapeutic strategies can be seriously considered.

## 1 Introduction

Periodontal disease, such as gingivitis and periodontitis, is a chronic inflammatory condition that compromises the integrity of the tooth-supporting tissues within the periodontal complex; these tissues encompass the gums, periodontal ligaments, root cementum and underlying bone structure (Gasner and Schure, 2024). Due to its prevalence, this disease represents a significant public health concern.

Conventional treatment of periodontitis is unlikely to eliminate all disease-causing pathogens. However, when it is systematically and accurately performed it has potential to establish a healthy ecosystem by altering the composition and numbers of microbial community and also, what is equally important, contribute to the maturation of the host immune response (Fragkioudakis et al., 2021). The current primary methods for treating periodontitis involve biofilm control, mechanical removal of plaque and tartar deposits, and antibiotic therapy. The main aim of this therapeutic strategy is to remove excess plaque and rebalance the composition of the oral microbiota (Tariq et al., 2012). However, the bacterial strains implicated in periodontal diseases have demonstrated growing antibiotic resistance (Gager et al., 2023; Rams et al., 2023; Ng et al., 2024); this presents a formidable challenge to conventional treatment modalities.

In light of the challenges associated with treating periodontal diseases, and the economic implications of managing both the disease and its systemic consequences, there is an urgent need for innovative research in the area, particularly initiatives aimed at exploring alternative therapeutic avenues capable of eliminating the bacterial agents responsible. One such promising approach is phage therapy, a therapeutic strategy harnessing bacteriophages (in short phages), viruses that exclusively target bacterial cells (Keen and Adhya, 2015). Phage therapy has garnered attention for its potential to address antibiotic resistance while effectively targeting bacteria residing within biofilms (Dicks and Vermeulen, 2024), known to play an important role in periodontal disease development (Abdulkareem et al., 2023). Another intriguing avenue for combating periodontal pathogens is bacteriotherapy (Huovinen, 2001; Pérez et al., 2016) in which predatory BALOs (Bdellovibrio and Like Organisms) bacteria may be used to selectively remove harmful microorganisms associated with periodontal disease while preserving beneficial species. The BALO cells attack pathogenic bacteria by attaching to the target cell surface, penetrating the periplasmic space using hydrolytic enzymes, multiplying and then a globular structure called “bdelloplast” performs target bacterial lysis. Such predatory bacteria have no specificity towards the bacterial host (broad prey range) or are highly specific towards the prey (Sockett and Lambert, 2004). Importantly, BALOs can also degrade biofilm (Dashiff and Kadouri, 2011; Mookherjee and Jurkevitch, 2022; Mun et al., 2023). Both bacteriophages and BALOs have been found to be effective against human and animal pathogens, and are hence promising candidates in the treatment of bacterial infections, especially those caused by multidrug-resistant (MDR) bacteria (Sun et al., 2017). This targeted approach may offer a nuanced alternative to broad-spectrum antimicrobial strategies, potentially minimizing disruptions to the oral microbiome.

Currently, both researchers and clinicians emphasize the important role played by the oral microbiome in preventing periodontal disease, and the need to maintain its equilibrium. It is equally important to select specific aims for new forms of targeted therapy (Haque et al., 2022); these can include the virulence factors of pathogenic bacteria and the regulatory systems controlling pathogenicity, or the factors responsible for the formation of biofilms, or even the biofilms themselves (Heras et al., 2015; García-Fernández et al., 2017; Rudkin et al., 2017; Tan et al., 2018; Fleitas Martínez et al., 2019).

This review aims to consolidate existing research on the application of phages and predatory bacteria in combating the bacterial pathogens associated with periodontitis. By exploring the efficacy, safety, and potential implications of phage therapy and bacteriotherapy in periodontal disease management, this review endeavors to contribute to the evolving landscape of periodontal therapeutics, offering insights into novel avenues for disease intervention and management.

## 2 Periodontal diseases—definition, symptoms, classification and health consequences

Periodontal disease most commonly occurs as a chronic inflammatory condition that damages the tissues supporting the teeth in the alveolus. These tissues include the gums, the periodontal ligament, the root cementum, and the bone. Such anatomical and histological changes in the periodontal tissues are clinically manifested by gingival bleeding, pain, exudate from the pockets, changes in shape (enlargement or thinning—recession), color and consistency of the gums, bad breath, loss of bone and connective tissue attachment. This can further lead to deepening of the periodontal pockets, the formation of recession (i.e., exposure of the tooth necks), tooth mobility, occlusal dysfunction, and ultimately, tooth loss (Oliver and Brown, 1993; Ong, 1998; Kapila, 2021).

In its advanced state, periodontal disease often impairs the private and social functioning of patients. The greater mobility of teeth, or their loss, prevents proper occlusion and biting, and leads to disturbances in proper phonation. Also, difficulties in chewing can disrupt food intake, negatively affecting the nutrition and general health of patients (Kapila, 2021). The destruction of bone tissue can also hinder subsequent prosthetic or implant-prosthetic treatment. Periodontal disease, the main cause of tooth loss after caries, can negatively affect chewing as well as the aesthetics, self-confidence and quality of life of patients. It also has a significant impact on general health, increasing the risk of systemic diseases such as peripheral arterial occlusive disease and associated hypertension: patients with advanced chronic periodontitis can present with a generalized inflammatory state, manifested by increased levels of C-reactive protein (CRP), fibrinogen, and pro-inflammatory cytokines (Mawardi et al., 2015; Bale et al., 2017). This may result in the formation of atherosclerotic plaques, thickening of the intima and media complex, endothelial dysfunction and increased vascular stiffness as well as heart attacks, strokes and other cardiovascular diseases. Periodontal disease is also believed to influence glycemia and thus the course and complications of diabetes. It has also been associated with lung disease, rheumatic disease, kidney disease, osteoporosis, low birth weight of newborns and predisposition to miscarriage (Seymour et al., 2007; Bourgeois et al., 2019; Liccardo et al., 2019; Sedghi et al., 2021).

Particular attention has focused on the positive correlation between inflammatory processes in the periodontium and the occurrence of circulatory system diseases (Table 1), including atherosclerosis, coronary artery disease and acute coronary syndromes, including heart attack (Sanz et al., 2000; Bourgeois et al., 2019; Liccardo et al., 2019). Indeed it has been found periodontal disease to be associated with a 25–72% greater incidence of coronary artery disease. Additionally, depending on age, a 25% greater risk of coronary artery disease was noted, and a 72% greater change was observed in men under 50 years of age, compared to people with healthy periodontium. Also, smokers with coexisting advanced periodontal disease are subject to an eightfold greater risk of cardiovascular disease. Furthermore, recent studies indicate that periodontal disease may also influence the development of neurodegenerative diseases (Dominy et al., 2019; Borsa et al., 2021); more specifically, a link has been reported between the bacteria that cause periodontal disease and the occurrence of Alzheimer’s disease (AD) and dementia (Maitre et al., 2021). Studies from the USA, Poland, Australia, and New Zealand report that the brains of patients with AD have been found to harbor *Porphyromonas gingivalis* DNA and gingipain antigens (Dominy et al., 2019). The increased presence of *P. gingivalis* in dental plaque also appears to favor the development of rheumatoid arthritis: an autoimmune disease in which anticitrullinated protein antibodies (ACPA) are produced against excess citrullinated proteins in the human body (Bourgeois et al., 2019) (Table 1). In summary, *P. gingivalis* increases the pool of citrullinated proteins in the host body; this overcomes immune tolerance and stimulates B lymphocytes to produce ACPA which then attack all citrullinated proteins. If the proteins have accumulated in the joints, this is where inflammation begins; over time, this inflammation becomes chronic, i.e., rheumatoid arthritis (Mikuls et al., 2014; Laugisch et al., 2016; Ceccarelli et al., 2019; Jenning et al., 2020; Li et al., 2022; Juárez-Chairez et al., 2024).

**Table 1:** Possible complications after periodontal diseases.

No.	Disease	Mechanism	Reference
1	Alzheimer disease	Secretion of gingipains by *P. gingivalis* promotes neuronal damage	Olsen et al. (2018), Dominy et al. (2019), Sansores-España et al. (2021), Cichońska et al. (2024)
2	Rheumatoid arthritis	Molecular mimicry; *P. gingivalis* produces an enzyme which has the ability to citrullinate proteins	Mikuls et al. (2014), Laugisch et al. (2016), Ceccarelli et al. (2019), Jenning et al. (2020), Juárez-Chairez et al. (2024)
3	Atherosclerotic changes	Periodontitis bacteria initiate a cascade of immunological response factors (increased levels of CRP, fibrinogen, and pro-inflammatory cytokines), which promotes the formation of atherosclerotic plaques	Chhibber-Goel et al. (2016), Huang et al. (2023), Rao and Kumar (2023)
4	Cardiovascular diseases, myocardial infarctions, coronary artery disease	The accumulation of atherosclerotic plaques leads to an increase in arterial pressure	Teles and Wang (2011), Guo et al. (2024), Tsai et al. (2024)
5	Diabetes	Increased production of inflammatory factors (e.g., TNF-α), which can act as insulin antagonists	Casarin et al. (2013), Gheonea et al. (2024)
6	Preterm birth	Oral plaque bacteria stimulate the inflammatory response, resulting in the release of pro-inflammatory cytokines, which enter the bloodstream and affect the fetus	Manau et al. (2008), Yang et al. (2022)

Gingipains, proteases degrading (among others) cytokines and downregulating the host’s immune response; citrullination, conversion of arginine residues in proteins into citrulline; CRP, C-reactive protein; TNF-α, tumor necrosis factor-alpha.

According to the current WHO definition, a healthy periodontium is defined as a state in which no periodontal inflammatory disease or clinical symptoms of previous disease can be observed (Lang and Bartold, 2018). In clinical practice, doctors distinguish between a primary, unaltered, healthy periodontium, i.e., pristine periodontal health with no histological or anatomical changes in the periodontium, and a clinically healthy periodontium, i.e., healthy, without signs of inflammation. This second case, more commonly encountered, is characterized by a reduced periodontium caused by previous disease, i.e., gingivitis, or healthy non-reduced tissue.

According to the new classification (World Workshop on the Classification of Periodontal and Peri-implant Diseases and Conditions) effective from 2017, periodontal diseases can be divided into gingivitis and periodontitis. Gingivitis is the initial and mildest form of the disease. However, if neglected and untreated, it can lead to further progressive degenerative changes in the tissues, finally resulting in the development of periodontitis. Gingivitis can be subdivided into a form caused by bacterial biofilm and a second form that is not. In contrast, three forms of periodontitis have been identified, viz. necrotizing periodontitis, periodontitis as a manifestation of systemic disease, and periodontitis, previously classified as chronic and aggressive (Caton et al., 2018). Periodontitis is also further classified into stages I through IV based on its degree of advancement (I—initial periodontitis, II—moderate periodontitis, III—severe periodontitis with the potential for additional tooth loss, IV—severe periodontitis with the potential for loss of dentition), and grades A to C based on its complexity (A—low rate of progression, B—expected progression, C—high risk of progression). When staging periodontitis, the main goals are to estimate the rate of periodontitis progression, guide the intensity of therapy, monitor the patient and determine the potential impact on systemic health (Alassy et al., 2021).

### 2.1 Epidemiology of periodontal disease

Regardless of their etiology and potentially severe local and systemic complications, periodontal disease (gingivitis, periodontitis) is one of the most common oral cavity conditions in humans. It is the main cause of tooth loss, along with caries and its complications. Due to its frequency and ubiquity, periodontal disease is considered a societal, pandemic disease. Periodontitis is the most common chronic inflammatory noncommunicable disease of humans. According to data originating from the Global Burden of Disease (GBD) database, 1.1 billion cases of severe periodontitis were prevalent globally in 2019, and an 8.44% increase in the age-standardized prevalence rate of severe periodontitis was observed.

In 2010, 10.8% of the global population was affected by severe periodontitis, while the majority of the adult population experienced mild or moderate periodontitis. This data placed periodontal diseases in sixth position among the most prevalent global conditions (Kassebaum et al., 2014); however, severe periodontitis alone, according to the Global Burden of Disease Study, was the eleventh most prevalent disease worldwide (GBD, 2018 Disease and Injury Incidence and Prevalence Collaborators, 2018; GBD, 2017 Oral Disorders Collaborators et al., 2020).

The World Health Organization (WHO) identifies periodontal disease as a pandemic condition, with alarming prevalence across different age groups and geographical regions. The 2022 global WHO report on oral health status estimated that oral diseases affect close to 3.5 billion people worldwide, two billion of whom suffer from dental caries in permanent teeth. In addition, periodontal diseases affect about 19% of the adult global population, accounting for over one billion cases. The same report for European countries indicates that over 50% of the European population may suffer from some form of periodontitis, with more than 10% suffering from its severe form, and the prevalence increases to 70–85% of the population aged 60–65 years (Jain et al., 2024). Between 1990 and 2019, the age-standardized prevalence rate of severe periodontitis increased worldwide by 8.44% (Chen et al., 2021; Kwon et al., 2021). This number significantly varies among different countries depending on many factors, including the nation’s hygiene self-awareness, social education, and the quality of dental care (Kim et al., 2009). A 2012 study in Poland found as much as 16% of the adult population in the largest cities suffers from an advanced form of periodontitis (Górska et al., 2012). Thus, the global burden of severe periodontitis is substantial and has significantly increased over the last three decades, presenting a major challenge for public health and prompting the search for new therapeutic solutions (Jain et al., 2024). Interestingly, the incidence of periodontal disease, including loss of attachment analysis, has been found to increase linearly with age (Billings et al., 2018).

### 2.2 Pathogenesis

Inflammatory periodontal diseases are widespread, chronic multifactorial disorders. A key role in their development is played by plaque deposition and the disruption of balance between the microbiological factor and the immune response of the host. A recent paper showed that microbial communities of different periodontal states changed asynchronously during biofilm reformation after its primary removal by supragingival scaling. It has been shown that bacteria such as *Abiotrophia* spp. and *Capnocytophaga* spp. might play an important role in determining the development of plaque biofilms (Fons-Badal et al., 2020; Li et al., 2023). Although such development appears to have a strong immunological-inflammatory basis (Bartold and Van Dyke, 2019), both congenital (genetic factors, sex) and acquired (smoking, stress, obesity, coexisting systemic diseases) risk factors also play a significant role. Through a combination of the direct, destructive effects of virulence factors secreted by the bacteria present in dental plaque, and the indirect activity resulting from the intensified, non-specific inflammatory response of the body to periopathogens, the gums, ligament and bone tissue in the mouth suffer increasing damage from progressing inflammation (Dahlen et al., 2019; Gao et al., 2020).

The pathophysiology of periodontitis is characterized by primary molecular pathways that ultimately lead to the activation of host-derived proteinases. These result in the destruction of the marginal fibers of the periodontal ligament, apical migration of the junctional epithelium, and consequently, the apical spread of the bacterial biofilm along the root surface. Gingivitis is a nonspecific inflammatory response to nonspecific bacteria, and hence is initiated by the bacterial biofilm, i.e., subgingivally-located nonspecific bacteria. In contrast, periodontitis is driven by ecological dysbiotic changes in the microbiome arising in response to products of inflammation and various antibacterial mechanisms. As such, there is no single, detectable, specific bacterial species responsible for destructive changes in the periodontal tissues. Despite this, strains belonging to the group of Gram-negative anaerobic rods are the most often found in active disease sites (Socransky et al., 1998). The bacteria involved in the development of periodontitis are primarily divided into 17 species belonging to the phyla *Bacteroidota* (formerly *Bacteroidetes*), *Bacillota* (formerly *Firmicutes*), *Fusobacteriota* (formerly *Fusobacteria*), *Pseudonomadota* (formerly *Proteobacteria*), *Spirochaetota* (formerly *Spirochaetes*), *Synergistota* (formerly *Synergistetes*), and candidatus *Saccharibacteria* (formerly known as Candidate Division TM7) (Dahlen et al., 2019; Oren and Garrity, 2021; Naud et al., 2023).

In gingivitis, the periodontal pocket depth is ≤3 mm, and during inflammation, its biomass is increased by the microbiota present around the gums and pocket. Additionally, inflammation also results in changes in the composition of the gingival biofilm, characterized by a decrease in the number of Gram-positive species (e.g., *Rothia dentocariosa*), and the increasing dominance of Gram-negative species (e.g., *Prevotella* spp., *Selenomonas* spp., *Fusobacterium nucleatum* ss. *vincentii*) (Carrouel et al., 2016). If left untreated, gingivitis can develop into periodontitis, with significantly greater tissue damage: the periodontal pocket reaches a depth of ≥4 mm, and the inflammatory state of the periodontium worsens, leading to bone defects. Significant changes in the subgingival biofilm composition also occur during the development of periodontitis; however, while Gram-negative bacterial species also generally predominate, the profile of the species differs from gingivitis (Hajishengallis, 2015).

**Figure 1:**
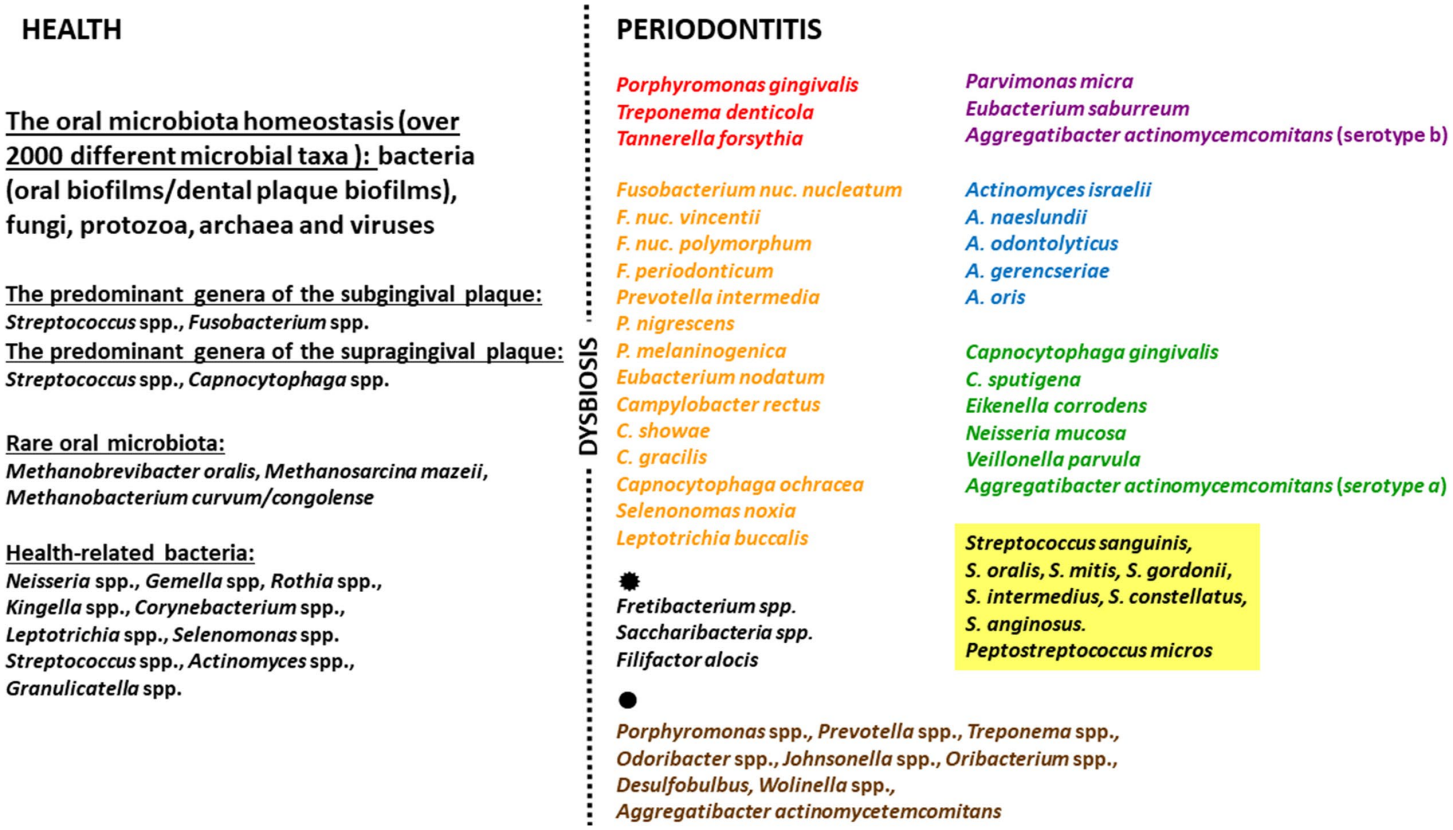
Model idea of the oral microbiome in periodontitis based on current literature data. The figure includes: the oral microbiota, the health-related species, the key pathogens in subgingival microbial complexes, other species in subgingival microbial complexes (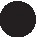), the novel periodontal pathogens (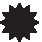).

One group of bacteria known to increase in number during the development of periodontitis is the so-called *red complex*, namely *P. gingivalis*, *Tannerella forsythia* and *Treponema denticola*, and these are recognized as the main etiological factors of this disease (Carrouel et al., 2016). These are accompanied by the orange, purple, blue, green, and yellow complexes. Although the concept of color complexes was introduced only at the beginning of widespread whole-genome sequencing era (Haffajee et al., 2008), it has been expanded since then and no changes in crucial pathogens were noticed. Studies suggest that *Actinomyces* species, bacteria of the green, yellow, orange, and red complexes, are associated with the long-term presence of periodontitis. Additionally, those belonging to the red complex (especially *P. gingivalis*) occur in greater numbers in areas of deep periodontal pocketing and bleeding (Teles et al., 2013). Other bacterial species have also been repeatedly identified in subgingival microbial complexes, including various novel periodontal pathogens, e.g.: *Fretibacterium* spp., *Saccharibacteria* spp. and *Filifactor alocis* (Abu Fanas et al., 2021; Ergün et al., 2023). Interestingly, reports suggest the existence of “health-related” species that prefer an anaerobic environment; however, their role in preventing the development of periodontal diseases requires more detailed research (Pérez-Chaparro et al., 2014; Abu Fanas et al., 2021; Antezack et al., 2023). A. model idea of the oral microbiome in periodontitis based on current literature data is presented in Figure 1.

Patients with healthy periodontium and good oral hygiene typically possess an eubiotic biofilm. Such biofilms are immature (i.e., constantly modified and removed by hygiene procedures) and do not present a threat to the host. These mainly contain Gram-positive cocci and streptococci, and other bacteria from the yellow and purple complexes (Pérez-Chaparro et al., 2014). In such conditions, the composition of the oral cavity microbiota can be regarded as being in a state of equilibrium; this balance is disrupted when oral hygiene deteriorates, and in this sense, periodontitis can be regarded as a dysbiosis in the bacterial biofilm (Van Dyke et al., 2020). Bacteria colonize the cervical areas of tooth crowns, forming a dental-gingival plaque that serves as a specific ecological niche for them, protecting against the action of antiseptic agents. When colonizing the oral cavity, pathogenic microorganisms use bacterial adhesins (e.g., lipopolysaccharide (LPS), fimbriae) to bind to receptors on the host cells (Dahlen et al., 2019; Brennan and Garrett, 2019).

The pathogenicity of bacteria associated with periodontitis is related to their production of multiple virulence factors, which affect their virulence, spread and destruction of host tissue. These include proteases, which stimulate the production and degradation of pro-inflammatory cytokines (IL-1β, IL-6, TNF-α) thus sustaining prolonged infections (Miyamoto et al., 2006), as well as cysteine proteases (gingipains), which directly destroy tissues and affect the immune response, and leukotoxins, which degranulate leukocytes. In addition, they secrete karilysins that release TNF-α from macrophages, thus degrading antibacterial peptides, as well as invasins that allow direct penetration of bacteria into host cells, and hydrogen sulfide that performs various roles, such as stimulating the release of pro-inflammatory cytokines IL-1β, IL-18 by monocytes, initiating apoptosis of gum fibroblasts and inducing the immune response. Some bacteria, including *P. gingivalis*, can also exhibit their virulence by evading phagocytosis by macrophages, thus avoiding the immune response (Werheim et al., 2020).

The stimulation of macrophages and neutrophils by bacterial virulence factors leads to excessive production of pro-inflammatory cytokines (TNF-α, IL-1β, IL-8, PG E2), which directly damage periodontal tissues (Garlet, 2010), and to increased levels of matrix metalloproteinases responsible for the destruction of collagen fibers (Pan et al., 2019). Among these, IL-1, IL-8, and TNF-α have the greatest influence on promoting neutrophil migration to sites of inflammation. In addition, IL-1 has been found to increase the expression of the receptor activator of nuclear factor-kappa B ligand on osteoblasts and T helper cells, which upregulate the maturation of osteoclasts and increase resorption of alveolar bone (Pan et al., 2019).

Hence, it is important to note that the etiology of periodontitis does not solely rely on the presence or absence of the short list of bacterial species given above; indeed, it has been proven that these bacteria are also detectable in healthy individuals (Ximénez-Fyvie et al., 2000). The key role is rather the significant change in the quantity and proportions of these periopathogens, as well as changes in their properties resulting from the interaction with immune response factors, and often with the coexistence of other congenital or acquired risk factors (Hajishengallis et al., 2012). These additional (beyond microbiological) risk factors can be divided into host-dependent local factors (root anatomy, tooth positioning, tooth crowding, dental fillings), host-dependent general factors (immune system, systemic diseases, genetic factors), and typically environmental factors (nutrition, medications, smoking, stress).

## 3 Classical treatment methods

**Figure 2:**
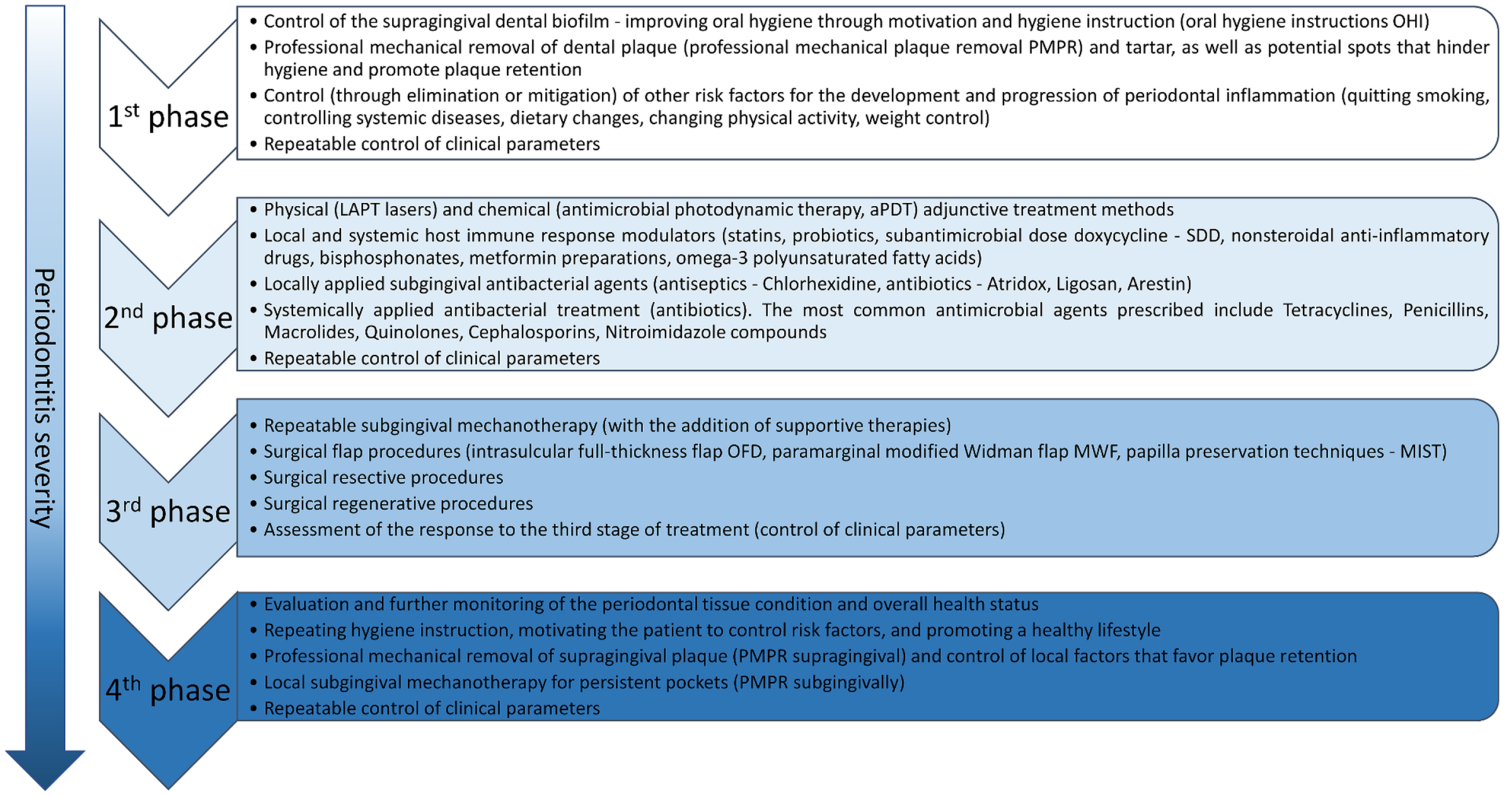
Summary of periodontitis treatment phases, and their numerous associated procedures.

Periodontal diseases associated with bacterial dental biofilm should first be subject to detailed periodontological diagnostics in accordance with the European Federation of Periodontology (EFP) algorithm (Sanz and Tonetti, 2019; Tonetti and Sanz, 2019) and treated in accordance with the previously-planned, staged therapy system (I–IV). Depending on the stage of the disease, the therapy consists of four phases, each of which includes numerous procedures (Sanz et al., 2020; Herrera et al., 2022). A summary of treatment phases and procedures for each phase are presented in Figure 2.

The first phase of therapy aims to change patient behavior, increase motivation to effectively remove supragingival dental plaque, and effectively control other risk factors. This phase must be implemented in every patient with periodontal inflammation, regardless of the degree and stage of the disease, and is a prerequisite for a successful response to the subsequent phases of treatment (van der Weijden and Slot, 2011; Chapple et al., 2018). The second phase of therapy, known as causal treatment, aims to reduce subgingival deposits (biofilm and calculus) and smooth the root surfaces by subgingival scaling and root planning (SRP), which generally refers to subgingival mechanotherapy using hand tools, ultrasonic, and sonic devices (van der Weijden and Slot, 2011; Chapple et al., 2018). The third phase, known as corrective treatment, is implemented in areas of the dentition where the therapeutic goals of the first and second stages of therapy were not achieved. The aim of this stage is to attain better access for the removal of subgingival deposits, and to repair, regenerate or resect any areas that significantly complicate the control of inflammation (e.g., changes within furcations, deep pockets with bone tissue loss). The fourth phase of therapy, known as supportive periodontal care (SPC), includes a combination of preventive and therapeutic procedures, repeated at various intervals and tailored to the patient’s needs. In this stage, mechanotherapy (PMPR) may be supplemented with adjunctive treatments to reduce periodontal inflammation, eliminate deposits, and stabilize the clinical outcomes, such as reduced pocket depth (PPD < 4 mm) and absence of bleeding on probing (BOP), ensuring long-term success. The second and fourth stages of periodontitis therapy have used the following approaches alongside professional mechanical plaque removal: physical means supporting subgingival mechanotherapy (lasers of various wavelengths, photodynamic therapy), chemical agents supporting mechanotherapy—antiseptics for use in the form of rinses and pastes and for subgingival administration (products containing chlorhexidine, triclosan copolymer, cetylpyridinium chloride) antibacterial substances (subgingivally or systemically).

The complex nature of the biofilm responsible for periodontal inflammation suggests that chemical antibacterial agents may be suitable for adjunctive treatment in every stage of therapy, beyond supragingival and subgingival mechanotherapy. Such agents include chlorhexidine, hydrogen peroxide, iodine preparations, essential oils, and even antibiotics. These agents can be used in mouthwash solutions, toothpastes, ointments, or gels for subgingival application. Antibiotic therapy is widely used in complex cases of advanced tissue destruction. The most commonly used antibiotics in the treatment of periodontitis are: amoxicillin, ampicillin, tetracycline, minocycline, doxycycline, erythromycin, clindamycin and metronidazole. Many observations and studies indicate that local, i.e., topical, administration of antibiotics and other drugs as adjuncts in the treatment of periodontal disease, offers more benefits than systemic antibiotic therapy (Herrera et al., 2008; Sholapurkar et al., 2020). In the short term, such treatment can yield a reduction in pocket depth (PPD), a reduction in BOP, and an improvement in the clinical attachment level (CAL). However, maintaining these results in long-term observations (>12 months) continues to be problematic, as does achieving the controlled release and the proper concentration of antiseptic or antibiotic agents from inflamed periodontal pockets. Other challenges include potential sensitization, the cost of preparations, and the limited availability of some products such as Periochip (Chlorhexidine gluconate), Atridox (active substance Doxycycline) and Arestin (active substance Minocycline) in European countries (Matesanz-Pérez et al., 2013). In addition, such treatment may also give rise to antibiotic resistance, as well as various allergies (like Ligosan -tetracycline, Gelcide -piperacillin and tazobactam) and systemic side effects (e.g., increased liver enzyme activity after using Doxycycline).

Summing up, periodontal diseases associated with bacterial dental biofilm require detailed diagnostics following the EFP algorithm and a staged therapy approach (Figure 2). The therapeutic strategy is multidimensional—encompassing both mechanical and chemical methods, applied in a specific order and frequently customized to meet the individual needs of the patient. However, maintaining long-term results and controlling the release and concentration of antiseptic or antibiotic agents pose challenges, along with potential antibiotic resistance and side effects.

## 4 Treatment efficacy issues—antibiotic resistance

Increasing numbers of bacteria demonstrate antimicrobial resistance (AMR), and as they are not sensitive to antibiotics, their eradication cannot be achieved (Salam et al., 2023). AMR can be classified as MDR, XDR or PDR depending on the nature of a given antibiotic-resistant strain or isolate. MDR refers to acquired non-susceptibility to at least one agent in three or more antimicrobial categories, XDR indicates non-susceptibility to at least one agent in all but two or fewer antimicrobial categories (i.e., bacterial isolates remain susceptible to only one or two categories), and PDR represents non-susceptibility to all agents in all antimicrobial categories (Magiorakos et al., 2012). Bacteria associated with periodontitis exhibit a wide spectrum of resistance to many antibiotics used in therapy. A comprehensive meta-analysis of the available literature on this subject was conducted by Abe et al. (2022). Table 2 details the antibiotic resistance profiles of various periodontal pathogens presented in the aforementioned publication. While we will not discuss each example provided, the data presented offer insight into the magnitude of the issue of antibiotic resistance among periodontal pathogens. Interestingly, these studies specify particular resistance genes responsible for the observed resistance. The authors emphasize that the most frequently cited genes in the analyzed studies are associated with resistance to erythromycin (*erm*), *β*-lactam antibiotics (*bla*_cfxA_), and tetracycline (*tet*) (Abe et al., 2022).

**Table 2:** Antibiotic resistance in periodontal pathogens according to Abe et al. (2022).

No.	Pathogen	Resistance
1	*Actinobacillus actinomycetemcomitans*	AMX, AXL, AZI, CFM, CFZ, CIP, CLI, CRX, CXE, CZD, DOX, MOX, MTZ, PEN, TET
2	*Actinomyces naeslundii*	AMP, AZI, CLI, DIC, MTZ, PEN, TET
3	*Alloprevotella* spp.	AMX, CFM, CLR, ERY, KAN, STR, TET
4	*Anaerococcus* spp.	AMX, CFM, CLR, ERY, KAN, STR, TET
5	*Bifidobacterium* spp.	AMX, CFM, CLR, ERY, KAN, STR, TET
6	*Campylobacter rectus*	CLI, DOX, SPI, TIN
7	*Capnocytophaga* spp.	AMX, AXL, CFM, CFM, CLI, CZD, ERY, KAN, STR
8	*Citrobacter freundii*	AMX, CEF, CLR, IMI
9	*Clostridium* spp.	AMP, PEN
10	*Dialister* spp.	AMX, CFM, CLR, ERY, KAN, STR, TET
11	*Eikenella corrodens*	AMP, AZI, CIP, CLI, DIC
12	*Enterobacter aerogenes*	AMX, CEF, CLR, IMI
13	*Enterobacter cloace*	AMI, AMP, AXL, CFM, CTZ, CZD, GEN, KAN
14	*Enterococcus avium*	AMX, AZI, CEF, CFM, CLI, CLR, IMI
15	*Enterococcus faecalis*	AMX, CLI, DOX, MTZ
16	*Enterococcuss* spp.	AMX, CIP, ERY, GEN, TEI, VAN
17	*Erwinia* spp.	AMX, AXL, AZI, CFM, CFZ, CIP, CLI, CRX, CXE, CZD, DOX, MOX, MTZ, PEN, TET
18	*Escherichia coli*	AMI, AMP, AMX, AXL, CEF, CFM, CLR, CTZ, CZD, GEN, IMI, KAN
19	*Fusobacterium nucleatum*	AMX, CLI, DOX, SPI, TIN
20	*Granulicatella adaicens*	AMP, AZI, CEM, CFM, CIP, CLA, CLI, DIC, ERI, LEV, LIZ, MTZ, OFX, PEN, TET, VAN
21	*Granulicatella elegans*	AZI, CFM, CLI, PEN, TET, ERI, CEM, CLA, LEV, LIZ, OFX, VAN
22	*Hafnia alvei*	AMI, AMP, AXL, CFM, CTZ, CZD, GEN, KAN
23	*Klebsiella oxytoca*	AMI, AMP, AXL, CFM, CTZ, CZD, GEN, KAN
24	*Klebsiella ozaenae*	AMX, CEF, CLR, IMI
25	*Klebsiella pneumoniae*	AMI, AMP, AXL, CTZ, CZD, GEN, KAN
26	*Leptotrichia* spp	AMX, CFM, CLR, ERY, KAN, STR, TET
27	*Morganella* spp.	AMX, CFM, CLR, ERY, KAN, STR, TET
28	*Olsenella* spp.	AMX, CFM, CLR, ERY, KAN, STR, TET
29	*Parvimonas micra*	AMX, CLI, DOX, MTZ, SPI, TIN
30	*Peptostreptococcus* spp.	AMX, CFM, CLR, ERY, KAN, STR, TET
31	*Porphyromonas gingivalis*	AMX, AZI, CLI, DOX, MTZ, SPI, TET, TIN
32	*Prevotella buccae*	AZI
33	*Prevotella denticola*	AZI
34	*Prevotella intermedia/nigrescens*	AMP, AMX, AZI, CLI, DOX, MTZ, PEN, SPI, TET, TIN
35	*Propionobacterium* spp.	AMP, PEN
36	*Pseudomonas aeruginosa*	CEF, CLR, IMI, TET
37	*Pseudomonas fluorescens*	CEF, CFM, CLR, IMI, TET
38	*Raoutella* spp.	AMX, CEF, CFM, CLR, IMI, TET
39	*Rothia dentocariosa*	AMX, AXL, AZI, CLI, DIC, MTZ
40	*Serratia liquefaciens*	AMI, AMP, AXL, CFM, CTZ, CZD,GEN KAN
41	*Serratia marcescens*	AMI, AMP, AXL, CFM, CTZ, CZD,GEN KAN
42	*Serratia odorifera*	AMI, AMP, AXL, CFM, CTZ, CZD,GEN KAN
43	*Shigella* spp.	AMI, AMP, AXL, CFM, CTZ, CZD, GEN, KAN
44	*Staphylococcus aureus*	AMP, AMX, AZI, CEF, CEM, CFM, CLA, CLI, CLR, CRX, DOX, ERI, ERY, IMI, LEV, LEV, LIZ, MTZ, OFX, PEF, PEN, TET, VAN
45	*Staphylococcus haemolyticus*	AMX, CEF, CLR, IMI
46	*Streptococcus anginosus*	AZI, CFM, CLI, ERI, CEM, CLA, LEV, LIZ, OFX, VAN
47	*Streptococcus constellatus*	AMX, AZI, CEM, CFM, CLA, CLI, DOX, ERI, LEV, LIZ, MTZ, OFX, SPI, TIN, VAN
48	*Streptococcus gordonii*	AZI, CEM, CFM, CLA, CLI, ERI, LEV, LIZ, OFX, VAN
49	*Streptococcus hyointestinalis*	CEM, CLA, ERI, LEV, LIZ, OFX, VAN
50	*Streptococcus intermedius*	AZI, CIP, DOX, MTZ, SPI
51	*Streptococcus mitis*	AMP, AZI, CEM, CFM, CLA, CLI, ERI, LEV, LIZ, OFX, PEN, TET, VAN
52	*Streptococcus mutans*	AZI, CEM, CFM, CLA, CLI, ERI, LEV, LIZ, OFX, PEN, VAN
53	*Streptococcus oralis*	AMP, AZI, CEM, CFM, CLA, CLI, ERI, LEV, LIZ, OFX, PEN, TET, VAN
54	*Streptococcus parasanguinis*	AZI, CEM, CFM, CLA, CLI, ERI, LEV, LIZ, OFX, TET, VAN
55	*Streptococcus pluranimalium*	AMP, AZI, CEM, CLA, CLI, ERI, LEV, LIZ, OFX, VAN
56	*Streptococcus pneumoniae*	AMX, AZI, CEF, CLI, CLR, IMI
57	*Streptococcus salivarius*	CEF, CLR, IMI
58	*Streptococcus sanguinis*	AMP, AZI, CEM, CFM, CLA, CLI, ERI, LEV, LIZ, OFX, PEN, TET, VAN
59	*Streptococcus sinensis*	AZI, CEM, CFM, CLA, ERI, LEV, LIZ, OFX, VAN
60	*Streptococcus thoraltensis*	AMP, CEM, CFM, CLA, ERI, LEV, LIZ, OFX, VAN
61	*Streptococcus tigurinus*	CEM, CLA, ERI, LEV, LIZ, OFX, VAN
62	*Tannarella forsythia*	AMX, AZI, CLI, DOX, MTZ, SPI, TIN
63	*Veillonella* spp.	AMX, CFM, CLI, CLR, DOX, ERY, KAN, MTZ, STR, TET

AMI, amikacin; AMP, ampicillin; AMX, amoxicillin; AXL, −amoxicillin + clavulanic acid; AZI, azithromycin; CEF, cefalotin; CEM, cefepime; CFM, cefotaxime; CIP, ciprofloxacin; CLA, clarithromycin; CLI, clindamycin; CLR, chloramphenicol; CTZ, cotrimoxazole; CXE, ceftriaxone; CZD, ceftazidime; DIC, dicloxacillin; DOX, doxycycline; ERY, erythromycin; GEN, gentamicin; IMI, imipenem; KAN, kanamycin; LEV, levofloxacin; LIZ, linezolid; MTZ, metronidazole; OFX, ofloxacin; PEF, pefloxacin; PEN, penicillin; SPI, spiramycin; STR, streptomycin; TET, tetracycline; TIN, tinidazole; VAN, vancomycin.

In most patients (68%) suffering from treatment-resistant (refractory) periodontitis, a number of bacteria produce *β*-lactamases, which can hydrolyze β-lactam antibiotics, such as penicillins, cephalosporins, monobactams and carbapenems (Handal et al., 2003). This is often the determining factor for therapeutic failures in such antibiotic therapy. Studies have reported β-lactamase production by various periodontal pathogens including *Porphyromonas*, *Prevotella*, and *Fusobacterium* species (Bernal et al., 1998; Handal et al., 2003; Abe et al., 2022). Other studies have reported the isolation of resistant pathogens from the inflammatory state of periodontal pockets in over 70% of patients with clinically-confirmed and most commonly-occurring chronic periodontitis (Rams et al., 2014a, 2014b). In such cases, the most resistant strains were *P. gingivalis*, *Prevotella* (*P. intermedia* or *P. nigrescens*), as well as *A. actinomycetemcomitans* and *Streptococcus constellatus*. *In vitro* studies have found bacterial strains to demonstrate resistance mainly to doxycycline, but also to amoxicillin, clindamycin, or metronidazole (Feres et al., 2002; Rams et al., 2014a, 2014b). A recent study on samples from German dental practices and hospitals found that *Staphylococcus* spp. and *Streptococcus* spp. can be common pathogens associated with periodontal diseases and exhibit significant resistance to a wide spectrum of antibiotics, with over 17% of strains not susceptible to macrolides and clindamycin (Meinen et al., 2021). Studies also show that nearly half of bacteria isolated from oral samples taken from children (such as *P. gingivalis*, *P. intermedia*, and *P. nigrescens*) contained genes conferring resistance to tetracycline and/or erythromycin, as well as to ampicillin and penicillin (Sanai et al., 2002; Ready et al., 2003). In addition, *A. actinomycetemcomitans*, *P. gingivalis*, and *T. forsythia*, commonly detected in bacteriological tests of patients with periodontal disease, often show resistance to amoxicillin, azithromycin, and metronidazole; however, studies have found moxifloxacin to have effective bactericidal action (Ardila and Bedoya-García, 2020).

Some reports also highlight the growing problem of drug resistance among bacteria causing periodontal diseases (BDJ Team, 2018). Numerous studies on the bacterial red complex report an increase in the numbers of isolates displaying resistance to some second-choice antibiotics. However, no significant differences in sensitivity were found against amoxicillin or metronidazole—two antibiotics frequently used in treating periodontitis (Kleinfelder et al., 1999; Ardila et al., 2010, 2023; Rams et al., 2014a; Jepsen et al., 2021). Generally, *P. gingivalis*, *P. intermedia*, *P. denticola*, *P. melaninogenica*, *F. nucleatum*, *T. forsythia*, *A. actinomycetemcomitans*, *S. constellatus*, *S. intermedius*, and *Parvimonas micra* have demonstrated a significant prevalence of antibiotic-resistant isolates. The highest frequency of resistance was observed for amoxicillin, clindamycin, and metronidazole, and the obtained antibiotic sensitivity profile appears to vary according to geographical region and patient age group (Ng et al., 2024).

Little research has been performed on the presence of specific resistance genes and their location in the genome of bacteria responsible for periodontal diseases. Research conducted in Brazil with the participation of 110 patients showed the presence of antibiotic-resistant genes (ARGs) in over 70% of participants. The following genes were detected: *erm*, *bla*_TEM_, *mecA*, *pbp2B*, and *aac(6′)*; these confer the macrolide-lincosamide-streptogramin B phenotype, or resistance to erythromycin, β-lactams and aminoglycosides (Alcock et al., 2019). No genes coding resistance to carbapenems or metronidazole were detected (Almeida et al., 2020). Another study examined the effects of periodontitis and SRP treatment on the performance of ARGs and metal-resistant genes (MRGs) in the dental plaque microbiota. After treatment, the number of ARGs and MRGs in dental plaque increased and the composition of the ARGs and MRGs profiles was significantly altered. Resistance genes to bacitracin, β-lactam, macrolide-lincosamide-streptogramin and tetracycline, as well as genes responsible for the MDR phenotype have been identified. Additionally, iron, chromium, and copper resistance genes were noted. The co-occurrence of ARGs and MRGs indicated that a co-selection phenomenon exists in the resistomes of dental plaque microbiota (Kang et al., 2021).

The task of keeping pace with new and more sophisticated mechanisms associated with emerging bacterial resistance is complicated by the fact that unfortunately, no new groups of antibiotics have emerged in the last 30 years that could be effective in the adjunct chemical therapy for treating periodontal diseases (Durand et al., 2019). This heralds the inevitable loss of antibiotics as the most useful and effective in treating gingivitis and periodontitis, prompting the search for new therapeutic approaches (Kaźmierczak et al., 2014).

Additionally, the development of periodontal disease is associated with the advancement and maturation of bacterial biofilms. Research indicates that biofilm is characterized by much higher resistance to antimicrobials compared to free-living bacteria (Wang et al., 2002; Flemming et al., 2016; Bowler et al., 2020). Indeed, it has been reported that bacteria cells within a biofilm can be 1,000-fold more resistant to antibiotics than planktonic cells (Donlan, 2000). The basis of this phenomenon is the diversity of the oral biofilm, referred to as the biofilm phenotype; this results from various interactions between the constituent bacteria (e.g., metabolism, horizontal gene transfer, adaptation mechanisms, tolerance to environmental threats, quorum sensing), which create optimal conditions for growth and multiplication (Mahuli et al., 2020). It is believed that biofilm formation offers the greatest protection to bacteria through the creation of an extracellular polymeric substance (glycocalyx) preventing effective antibiotic penetration, and the existence of persistent cells that survive after antibiotic therapy (Bowler et al., 2020).

Summarizing, the increasing AMR in bacteria poses significant challenges in treating periodontal diseases. Biofilms in periodontal disease are highly resistant to antibiotics, often requiring higher doses for effectiveness. Many periodontal pathogens produce β-lactamases, rendering β-lactam antibiotics ineffective, and exhibit resistance to other commonly used antibiotics like doxycycline, amoxicillin, clindamycin, and metronidazole. Studies highlight the presence of ARGs in bacteria associated with periodontitis, complicating treatment. Despite the urgent need for new antibiotics, no new groups have been developed in the past 30 years, necessitating alternative therapeutic strategies.

## 5 Alternative therapies

The current basic methods of treating periodontitis are based on the mechanical removal of plaque and calculus deposits and/or antibiotic therapy. The main aim of such strategies is to remove excess amounts of material and rebalance the composition of the oral microbiota. Due to the prevalence of periodontitis, the number of systemic complications associated with the disease, and the urgent need to limit the use of antibiotics, there is a need to develop and implement alternative treatments. The review will therefore describe potential alternative strategies for combating pathogenic microorganisms based on the use of bacteriophages and predatory bacteria.

### 5.1 Bacteriophage therapy

Bacteriophages (phages) are the most abundant and diverse group of biological entities found in nature. Their numbers exceed those of bacteria by tenfold, implying that theoretically, every bacterium has at least one phage capable of infecting it (Weinbauer, 2004; Mushegian, 2020). Phages, as obligate intracellular parasites, infect bacterial cells by recognizing specific receptors on their surface; they then enter the cell, replicate and release progeny particles, often leading to the destruction of the cell. This ability of phages to specifically infect and destroy bacteria, including pathogenic ones, is harnessed for therapeutic purposes as *phage therapy* (Keen and Adhya, 2015).

In the early days of phage therapy in humans, phages were used to treat different diseases. Many of these therapies are currently in clinical trials stage with the most promising results for the urinary tract treatment (Table 3).

**Table 3:** Reported diseases treated by phage therapy in humans.

No.	Disease	Etiological factor	Reference
1	Dysentery Typhoid Skin infections Surgical wounds Urinary tract infections Sepsis Peritonitis Otitis externa	na	Wittebole et al. (2014)
2	Pneumonia Meningitis Osteomyelitis Postoperative infections in cancer patients	na	Sulakvelidze et al. (2001)
3	Burns*	*P. aeruginosa*	Jault et al. (2019)
4	Sepsis in patients with acute kidney injury*	*P. aeruginosa*	Jennes et al. (2017)
5	Mycobacterium diseases*	*Mycobacterium* spp.	Dedrick et al. (2019)
6	Urinary tract infections*	*S. aureus* *E. coli* *Streptococcus* spp. *P. aeruginosa* *P. mirabilis*	Ujmajuridze et al. (2018)
7	Diarrhea*	*E. coli*	Sarker and Brüssow (2016), Sarker et al. (2017)
8	Periprosthetic joint infections*	*S. aureus*	Ferry et al. (2018)
9	Leg ulcers*	*S. aureus* *E. coli* *P. aeruginosa*	Rhoads et al. (2009)
10	Ear infections*	*P. aeruginosa*	Wright et al. (2009)

na, not applicable (data not reported in study); *, during clinical trials.

These trials utilized a single phage or so-called *cocktails* of at least two kinds of phages, often administered in combination with antibiotics to obtain observed additive or synergistic effects (Comeau et al., 2007). While some of the current human clinical trials have already moved on to Phase III, e.g., treatment for tonsillitis and urinary tract infections, most remain in Phase I or Phase II (Yang et al., 2023). It should be mentioned that bacteriophage therapy has been granted Emergency Use Authorization (EUA) by the U.S. Food and Drug Administration (FDA). It allows the compassionate use of experimental phage therapy on a case-by-case basis only for patients who have no other therapeutic options (Hitchcock et al., 2023).

The use of bacteriophages has a number of advantages. Most phages are highly specific to bacteria, infecting only specific species or even strains, without affecting the natural microbiota. Moreover, they replicate within host cells as long as their host remains present, which theoretically suggests that a single dose should be sufficient, eliminating the need for a booster. Furthermore, as the mechanism of action of bacteriophages differs from that of antibiotics, bacterial resistance to a given antibiotic does not confer resistance to bacteriophages, which makes them effective in treating diseases caused by MDR bacteria (Loc-Carrillo and Abedon, 2011). In addition, bacteriophages are composed mainly of proteins and nucleic acids, which are non-toxic to humans and animals (Loc-Carrillo and Abedon, 2011). Furthermore, phage therapy is associated with lower production costs than antibiotic production (Loc-Carrillo and Abedon, 2011).

However, despite these advantages, it is possible for bacteria to develop resistance to phages, which is one of the major drawbacks of phage therapy (Oechslin, 2018). The most common mechanism of resistance is a spontaneous mutation in the gene encoding the receptor recognized by a specific phage (Schroeder et al., 2018). Another mechanism, called CRISPR-Cas (clustered regularly interspaced short palindromic repeats), is an adaptive immune mechanism responsible for recognizing and degrading foreign DNA (including phage genetic material), thus acquiring permanent immunity to reinfection (Mohamadi et al., 2020). Thorough, whole-genome sequencing of red-complex bacteria (*P. gingivalis*, *T. forsythia* and *T. denticola*) revealed the presence of functional CRISPR-Cas systems which may provide efficient phage immunity (Chen and Olsen, 2019; Yadalam et al., 2023). Despite this, it has been shown that phages may have their own “anti-bacterial” CRISPR weapon (Seed et al., 2013) or they can suppress bacterial CRISPR immunity using protein-based inhibitors, RNA-based anti-CRISPRs, or solitary repeat units, thus fueling the constant evolutionary arms race between bacteria and phages (Camara-Wilpert et al., 2023). In addition, in some cases, phage treatment has resulted in the production of specific anti-phage antibodies, thus reducing the effectiveness of prolonged therapy (Łusiak-Szelachowska et al., 2014).

The efficiency of phage preparation and production is also important, as it depends directly on the way the host is cultivated. Although it is usually a quick and simple process, propagation and isolation of suitable phages can be complicated when the bacterial culture is more demanding. The clinical aspects of phage therapy for various body lesions, except for the oral cavity, have been recently reviewed by Hitchcock et al. (2023) and Fowoyo (2024).

#### 5.1.1 Bacteriophage therapy of periodontal pathogens

While phage therapy has already been found to be effective against many infections, few studies have focused on its use in the treatment of oral diseases. Nevertheless, phages have been shown to be effective against root canal infections associated with *Enterococcus faecalis*: a foodborne pathogen associated with many diseases which may also play a role in periodontal health (Paisano et al., 2004; Vidana et al., 2011; Khalifa et al., 2016). However, to carry out phage therapy for periodontitis, it is necessary to isolate phages capable of infecting bacteria strongly associated with this disease, i.e., anaerobic bacteria belonging mainly to the genera *Aggregatibacter*, *Fusobacterium*, *Porphyromonas*, *Prevotella*, *Tannerella*, *Treponema*, and *Veillonella*, as well as various new and emerging periodontal pathogens, such as *F. alocis*, *Slackia exigua* or *Eubacterium saphenum* (Aruni et al., 2015; Vieira Colombo et al., 2016; Antezack et al., 2023; Shen et al., 2023). One of the most demanding aspects of using phage therapy against these bacteria is that all listed bacteria species are difficult to cultivate in laboratory conditions. Furthermore, *in situ* therapy may be complicated by their growth in the form of dental plaque, i.e., a multispecies biofilm that is highly resistant to antimicrobials (Hall and Mah, 2017). A recent review examined the issue of using bacteriophage therapy against bacterial biofilms; however, oral cavity biofilms were not specifically addressed (Doub, 2020; Grooters et al., 2024). Examples of phage therapy against periodontitis-causing bacteria summarized in Table 4.

**Table 4:** Examples of phage therapy against periodontitis causing bacteria.

No.	Phage	Bacterial pathogen	Outcome	References
1	Fnpf02	*F. nucleatum* subsp. vincentii *F. nucleatum* subsp. polymorphum	Efficacy varied and the lysis of bacteria occurred very slowly	Machuca et al. (2010)
2	фFunu1 фFunu2	*F. nucleatum* subsp. animalis (strain 7–1) *F. vincentii* *F. polymorphum*	Both phages were unable to infect bacteria	Cochrane et al. (2016)
3	FNU1	*F. nucleatum*	Kills cells of the single-species biofilm *F. nucleatum* ATCC 10953 with 70% efficacy; has a potential proved by genetic analysis to be useful in more complex system such as polymicrobial dental plaque	Kabwe et al. (2019)
4	P1	*F. nucleatum* *B. subtilis* *B. thuringiensis* *E. coli* *C. butyricum*	The authors did not use these phages in oral diseases therapy	Zheng et al. (2019)
5	P2	*F. nucleatum*	The authors did not use these phages in oral diseases therapy	Zheng et al. (2019)
6	JD-Fnp1 JD-Fnp2 JD-Fnp3 JD-Fnp5	*F. nucleatum*	Lytic phages	Wang et al. (2022)
7	JD-Fnp4	*F. nucleatum*—several clinical isolates and two pathogenic *F. nucleatum* standard strains, (ATCC 25586 and ATCC 23726)	Phage remains relatively stable at a normal body temperature of 37°C, and achieves very high adsorption rate—more than 80% in a very short time 5 min; genome analysis confirmed the lack of any virulence factors or antibiotic resistance genes	Wang et al. (2022)
8	фtd1	*T. denticola* ATCC 35405	No further data on phage фtd1 was found	Mitchell et al. (2010)
9	Aabф01	*A. actinomycetemcomitans* PAA005 serotype b	Ineffective against *A. actinomycetemcomitans* PAA005 serotype c or numerous other periopathogens, for example *P. gingivalis*, *P. intermedia*, *F. nucleatum*; Aabф01 phage lysed bacterial cells in planktonic form during the early exponential growth phase but did not eliminate the bacterial culture completely; further characterization of the virus and its use as a potential therapeutic agent was planned, but no new reports have been published to date	Castillo-Ruiz et al. (2011)
10	S1249	*A. actinomycetemcomitans*	Pseudolysogenic phage; its infection increase sensitivity of host bacterium to human serum	Tang-Siegel (2023)
11	ϕAa17	*A. actinomycetemcomitans*	Similar to S1249	Stevens et al. (1982)
12	A2	*Neisseria* spp. (from dental plaque and saliva)	The authors do not indicate the immune strains isolated during the study nor did they analyze the host range for specific periodontopathogens; no further research on phage A2 can be found	Aljarbou and Aljofan (2014)

The first step towards the phage therapy of periodontitis was the research carried out by Machuca et al. (2010) on *F. nucleatum*. This bacterium is a common member of the gut and oral microbiota and has recently been associated with numerous problems such as inflammatory bowel disease, adverse pregnancy outcomes and colorectal cancer; it has also been implicated in periodontal disease, where it forms a backbone of the polymicrobial dental plaque (Han, 2015; Brennan and Garrett, 2019; Șurlin et al., 2020). Machuca et al. (2010) isolated a phage infecting *F. nucleatum* from saliva samples of healthy individuals. Three subspecies (*F. nucleatum* subsp. nucleatum, *F. nucleatum* subsp. vincentii, and *F. nucleatum* subsp. polymorphum) were targets of the Fnpf02 phage, but its efficacy varied and the lysis of bacteria occurred very slowly. The authors speculate that these observations may have resulted from lysogenic cycles, with the life cycles of this phage being undetermined at that point, or from the absence of certain culture conditions. No further research on Fnpf02 has been published, suggesting that no promising therapeutic effects were obtained. The literature also contains previous reports on the identification of phages infecting oral bacteria, but without indicating a specific therapeutic target (Szafrański et al., 2017).

Another study obtained phages фFunu1 and фFunu2 specific to *F. nucleatum* subsp. animalis (strain 7-1) (Cochrane et al., 2016). The bacterial strain was initially isolated from the colon of a Crohn’s disease patient, and later studies found it to contain chromosomal features common to many different *F. nucleatus* strains in the form of prophages (Wilde and Allen-Vercoe, 2023). The prophages were successfully isolated with mitomycin C and their genomic DNA sequences were analyzed. Unfortunately, both phages proved to be defective and unable to infect bacteria, including isolates representative of the *F. animalis*, *F. vincentii* and *F. polymorphum* subspecies (Cochrane et al., 2016); this was also observed in a later study, and assumed to be the result of the strong defenses (CRISPR) of the applied *Fusobacterium* strains (Wilde and Allen-Vercoe, 2023).

The first confirmed active lytic *F. nucleatum* phage, FNU1, was isolated from dental practice mouthwash: a drainage sample from dental chairs (Kabwe et al., 2019). It demonstrated the ability to kill cells of the single-species biofilm *F. nucleatum* ATCC 10953 with 70% efficacy. The genome of the FNU1 bacteriophage was fully sequenced and subjected to *in silico* analyses, which revealed a lack of homology to other known viral genomes. Although the bacterial biofilm responsible for the development of periodontitis is not a single species, such as the one used in this study, the authors suggest that FNU1 has the potential to be applied in more complex systems, for example, dental plaque during periodontitis. This is further supported by the fact that almost 8% of FNU1s ORFs may take part in the defense against bacterial anti-bacteriophage systems, and some additional genes may be involved in preventing abortive infections (Kabwe et al., 2019). Interestingly, Kabwe et al. (2021) published a the method for screening samples that may contain bacteriophages against oral pathogenic bacteria, which could be very useful for the use of phages in the treatment of oral diseases in the future.

Two other phages, P1 and P2, enable to infect *F. nucleatum* were identified and isolated by Zheng et al. (2019). For isolation, fecal samples from mice and saliva samples obtained from humans were used. P1 phages exhibited a wide antibacterial spectrum (*F. nucleatum*, *Bacillus subtilis*, *B. thuringiensis*, *E. coli* and *C. butyricum*). In contrast, P2 phages specifically inhibited the growth of *F. nucleatum*, leaving other bacteria completely unaffected. The authors did not use these phages in oral diseases therapy. Instead, they use P2 phages in modulation of the gut microbiota of mouse models of colorectal cancer augments. Moreover, the same group presented four other phages infecting *F. nucleatum* (Zelcbuch et al., 2020) which indicates that lytic phages against this bacterium occur quite frequently in nature.

The most recent study by Wang et al. (2022) presents a characterization of five new lytic phages of *F. nucleatum*; however, no significant similarity was found to FNU1 at the genome level. Four of these phages were isolated from saliva from healthy people (JD-Fnp1, JD-Fnp2, JD-Fnp3 and JD-Fnp4) and one (JD-Fnp5) was derived from fecal samples of a patient with colorectal cancer. Numerous biological and genetic analyses indicate that JD-Fnp4 may have the greater potential for use in clinical applications: it has the broadest host range, including several clinical isolates and two pathogenic *F. nucleatum* standard strains, (ATCC 25586 and ATCC 23726), it remains relatively stable at a normal body temperature of 37°C, and achieves very high adsorption rate (more than 80%) in a very short time (5 min). Importantly, genome analysis confirmed the lack of any virulence factors or antibiotic resistance genes among the five described phages, which is a crucial part of any safety assessment (Wang et al., 2022).

Another study also searched for species-specific bacteriophages for *T. denticola* ATCC 35405 (Mitchell et al., 2010). The search began with an analysis of the transcriptome of bacteria growing both in planktonic and in biofilm forms. The obtained material was found to contain regions with unusual nucleotide compositions. Genes encoding proteins with a similar amino acid sequence to known bacteriophage proteins (e.g., Bordetella BPP-1 phage or enterobacterial shigella-toxin-converting phages) were found to demonstrate increased expression. Further studies confirmed the presence of phage DNA in the culture superfiltrate, which suggested that active phage particles had escaped from the cell. These assumptions were validated by electron microscopy visualization of phage фtd1 (Mitchell et al., 2010). However, the aim of the work was not to search for phages and their further use, but rather to characterize *T. denticola* ATCC 35405 strain. No further data on phage фtd1 was found.

Substantially more information is available regarding the phages specific to *A. actinomycetemcomitans*, associated with localized aggressive periodontitis (Gholizadeh et al., 2017). However, most of the identified phages are temperate, and therefore not the first choice for therapy (Szafrański et al., 2017, 2019). The only currently-known virulent phage was phage Aabф01, isolated by Castillo-Ruiz et al. (2011) from saliva and wastewater samples taken from dental chair drainage. The phage was found to be effective against *A. actinomycetemcomitans* PAA005 serotype b associated with periodontitis but ineffective against serotype c or numerous other periopathogens, for example *P. gingivalis*, *P. intermedia*, *F. nucleatum*. The study showed that the Aabф01 phage lysed bacterial cells in planktonic form during the early exponential growth phase but did not eliminate the bacterial culture completely. To improve the effectiveness of the infection, the phage was mutagenized with UV light. The resulting mutant phage Aabф01-1 indeed lysed bacteria more effectively, even within a biofilm (99% efficacy). The authors did not specify which part of the phage DNA had been mutated, nor did they analyze the effects of the altered phage on other organisms in the oral cavity. The only phage feature addressed was its life cycle. UV treatment of *A. actinomycetemcomitans* PA005 did not promote bacterial lysis indicating that parental phage Aabφ01 was not a product of lysogen induction (Castillo-Ruiz et al., 2011). Further characterization of the virus and its use as a potential therapeutic agent was planned, but no new reports have been published to date. The other phage infecting *A. actinomycetemcomitans* is S1249. It was isolated by Tang-Siegel et al. (2023). The phage was described as pseudolysogenic and its infection increased sensitivity of host bacterium to human serum (Tang-Siegel, 2023). Despite the fact that phage S1249 is not a lytic phage, its presence in the bacteria-human immune system may influence the abundance of the pathogen.

The next phage identified by mitomycin C induction is ϕAa17 (Stevens et al., 1982). Interestingly, this phage was also induced by human factor which is human gingival fibroblasts (Stevens et al., 2013). Therefore, it could be concluded, similarly to S1249 phage, that presence of such lysogens could influence the pathogenic bacteria number in human oral infection.

Aljarbou and Aljofan (2014) isolated and characterized the first lytic phage (phage A2) infecting the genus *Neisseria* from dental plaque and saliva as a clinical material. *Neisseria* spp. is an oral commensal which generates an anaerobic niche for periodontopathogens (Liu et al., 2015). The structure of the phage was described by a combination of DNA sequencing, electron microscopy and bioinformatic analysis, and the host strain obtained from the same sample was assigned to *N. subflava* or *N. perflava* (Aljarbou and Aljofan, 2014). However, the authors do not indicate the immune strains isolated during the study nor did they analyze the host range for specific periodontopathogens. Although the indication of phage therapy against *Neisseria* spp. was implied, no further research on phage A2 can be found.

The diversity of oral bacteriophages and their impact on the biofilm formed by bacteria present in the mouth are presented in a recent review (Szafrański et al., 2021). Although more than 2,000 oral phages are believed to be capable of infecting members of the phyla *Actinomycetota* (formerly *Actinobacteria*), *Bacteroidota, Bacillota, Fusobacteriota, Pseudonomadota, Synergistota* and *Spirochaetota*, only a few phages infecting periodontopathogens have been studied so far (described above). Most of the presented data was obtained from metagenomic profiling accessible in the Integrated Microbial Genome/Virus (IMG/VR) database (Camargo et al., 2023). Interestingly, according to the IMG/VR, no lytic phages of *P. gingivalis* were found, and only four phages were predicted against *T. forsythia*, and 19 against *P. intermedia/Prevotella nigrescens* (Szafrański et al., 2021). In addition, two parallel investigations documenting the presence of numerous prophages in *P. gingivalis* genomes have been published, recently Gu et al. (2023) screened 90 *P. gingivalis* strains originating from different parts of the world with Prophage Hunter software and predicted a total of 69 prophages within 24 strains. Although 17 prophages were classified as “active,” the experimental work awaits to confirm these findings. Importantly, some of the phages carry genes that encode virulence factors or ARGs, and therefore these particles may be assumed as fitness factors in the pathogenicity of *P. gingivalis*. The second work, by Matrishin et al. (2023), analyzed the prophage profile of *P. gingivalis* (79 strains) even more deeply by combining the prediction of prophage regions with CRISPR spacers and the available prophage, ribosomal proteins and genome databases, resulting in phylogenetic analysis of identified phages. The authors found 24 different “full” temperate phages assigned to three clades among 26 strains. Aside from essential (structural and functional) viral genes the prophages carried LPS-modifying enzymes, proteins with signal sequence for general secretion systems and toxin-antitoxin systems. Interestingly, the gene annotation scheme used in this study did not identify any ARGs even for the same subset of strains. However, the authors presented evidence of prophage activity for the *P. gingivalis* ATCC 49417 strain employing full sequencing of phage005 DNA from liquid culture and electron microscopy (Matrishin et al., 2023). Other important questions such as host range, cell surface receptor, or impact on host colonization remain to be discovered. This would be the second phase of a member of the red-complex (together with the one from *T. denticola* Mitchell et al., 2010) characterized experimentally to a certain extent. However, it needs to be emphasized that phage therapy is established based on lytic phages not temperate. Therefore, further research or genetic modifications of identified phages is needed.

### 5.2 Bacteriotherapy—overview

Another interesting approach aimed at eradication or limitation of bacterial pathogens is bacteriotherapy. The use of predatory bacteria for periodontal treatment would allow the removal of only those species of microorganisms that are harmful to periodontal health, while not affecting the number of species exhibiting protective properties. This approach offers great potential, as the treatment directly addresses the disease while preserving the natural beneficial microbiota (Huovinen, 2001). Conventional therapies are much more invasive and require restoration of the microbial balance in the oral cavity.

Predation is a commonplace, natural interaction between predator and prey in which only the predator benefits. In this case, the predator is the carnivorous bacteria in a given ecosystem that live by the selective killing of certain species of bacteria, and using the decomposition products of the prey as nutrients for growth and multiplication. As such, interest has grown in the possibility of using them in the treatment of periodontitis caused by imbalances in the oral microbiome, particularly characterized by high proportions of Gram-negative bacteria. When using this form of control, it is important that the predatory bacteria do not show pathogenicity toward the human body; as such, the BALO (*Bdellovibrio* and like organisms) predatory bacteria were particularly promising candidates. Most of these species can attack both related and phylogenetically-distant Gram-negative bacteria. BALOs were first described in the 1960s and are by now the best-characterized group of predatory bacteria (Stolp and Starr, 1963).

Among these BALOs, *Bdellovibrio bacteriovorus* is the best known and most widely described. It exhibits a biphasic life cycle, consisting of an attack phase followed by a growth phase. During the former, the bacterium attaches itself to its prey, enters the periplasmic space and begins a replicative cycle, drawing nutrients from the cytoplasm. When the replicative cycle is complete, it ruptures the remnants of the host cell, releasing the progeny into the environment; hence, the cycle ends with the lysis of the host cell (Sockett and Lambert, 2004). Additionally, there is some evidence suggesting *B. bacteriovorus* may use an epibiotic predation strategy on Gram-positive bacteria like *S. aureus*, but data on this is limited (Waso et al., 2021).

Several studies have demonstrated the efficacy of BALOs in reducing bacterial burden during infection, and examined the safety of administration on various animal models. Much attention has been paid to MDR pathogens such as *Klebsiella pneumoniae*, *P. aeruginosa*, *Serratia marcescens*, or adherent-invasive *E. coli* (AIEC), to name a few (Shatzkes et al., 2015, 2016; Bonfiglio et al., 2020; Romanowski et al., 2024). Additionally, BALOs have been found to be successful in limiting Tier 1 select agent *Yersinia pestis* during infection in mouse lungs (Russo et al., 2019). However, in a study on a rat model, it was found that while intravenous application was not harmful, it did not cure *K. pneumoniae* infection, or prevent acute blood infection or the dissemination of the pathogen to other organs (Shatzkes et al., 2017).

#### 5.2.1 Bacteriotherapy in the oral cavity

Utilizing predatory bacteria in the oral cavity may be a challenging task. Firstly, the mouth does not provide the most favorable conditions for microorganisms. For example, the presence of saliva and an anaerobic environment can pose considerable difficulties to BALOs, both in terms of their viability and ability to predate on other bacteria. Nonetheless, research has demonstrated that this is possible (Table 5).

**Table 5:** Examples of the potential use of BALOs to control an oral bacterium.

No.	BALO	Oral bacterium	Outcome	References
1	*B. bacteriovorus* strains	*A. actinomycetemcomitans* *E. corrodens* *P. intermedia* *F. nucleatum*	Reduction of the planktonic growth of indicated oral pathogens	Van Essche et al. (2011)
2	*B. bacteriovorus* strain 109J	*A. actinomycetemcomitans E. corrodens*	Reduction of the planktonic growth of indicated oral pathogens	Dashiff and Kadouri (2011)
3	*B. bacteriovorus* strain 109J	*A. actinomycetemcomitans E. corrodens*	Efficiently elimination of both bacterial biofilms, even in the presence of saliva	Dashiff and Kadouri (2011)
4	*B. bacteriovorus* strain HD100	*A. actinomycetemcomitans*	BALO can penetrate the biofilm, there was a reduction of bacterial cells in both planktonic culture and biofilm	Van Essche et al. (2009)
5	*B. bacteriovorus* strain HD100	*F. nucleatum* *A. actinomycetemcomitans*	Elimination of the indicated bacteria *in vitro* and *ex vivo*	Loozen et al. (2015)
6	*B. bacteriovorus* strain HD100	*E. coli* *E. corrodens*, *A. actinomycetemcomitans*	Ability of preying on aerobic and microaerophilic bacterial species	Patini et al. (2019)
7	*B. bacteriovorus* strain HD100	Bacteria occurring in experimental periodontitis in rats	A decrease in the number of bacteria involved in the development of periodontitis *in vivo*: *P. intermedia*, *Peptostreptococcus micros*, *F. nucleatum*, *F. polymorphum*, *E. corrodens*, *E. nodatum*, *C. gracilis*, *C. sputigena*, and *V. parvula*-like species; reduction of bone loss, improve bone microstructure and protecting affected tissues, modulation of the immune response of periodontal tissues during periodontitis	Silva et al. (2019, 2023)

The first study evaluating the potential use of BALOs to control an oral bacterium was carried out in 2009. It examined the potential of *B. bacteriovorus* strain HD100 to counter the planktonic and biofilm forms of *A. actinomycetemcomitans*, a key pathogen associated with an aggressive form of periodontitis. In the first case, pure cultures of four strains of *A. actinomycetemcomitans* (ATCC 43718, ATCC 29523, ATCC 33384, JP2) were incubated with a suspension of *Bdellovibrio*, and prey survival was analyzed quantitatively by culture and qPCR. The study also assessed the effect of the BALO on a 2-day biofilm formed by *A. actinomycetemcomitans* 2,751 by scanning electron microscopy (SEM) and crystal violet staining over several time points. It was found that BALOs are partially effective in reducing *A. actinomycetemcomitans* in both planktonic culture and biofilm, but complete eradication was not achieved, and the biofilm structure was only partially destroyed. Nonetheless, it is important to note that *B. bacteriovorus* HD100 can penetrate the biofilm (the basic form of bacterial growth in the oral cavity), i.e., get through the matrix (EPS/ECM) and attack bacterial cells, which causes partial destruction of the biofilm structure (Van Essche et al., 2009).

The same group also assessed the efficiency of different BALO strains at reducing the planktonic growth of various oral pathogens, including *A. actinomycetemcomitans*, *E. corrodens*, *P. gingivalis*, *C. sputigena*, *P. intermedia* and *F. nucleatum*. The study indicated that the BALOs exhibited different prey spectra and various susceptibility of prey strains. The most potent predator was strain HD100, attacking four kinds of prey, and the most vulnerable pathogen was *F. nucleatum*, altered by four different predator strains. Two pathogens, viz. *P. gingivalis* and *C. sputigena*, demonstrated immunity to all tested *B. bacteriovorus* strains. Importantly, the findings indicated for the first time that BALOs can predate on selected strictly anaerobic bacteria (*F. nucleatum* and *P. intermedia*). However, it needs to be emphasized that the analyses were carried out in an aerobic environment suitable for *Bdellovibrio*, using planktonic cultures that do not accurately reflect the conditions in the subgingival plaque (Van Essche et al., 2011).

The potential of *B. bacteriovorus* strain 109 J to predate on various *A. actinomycetemcomitans* and *E. corrodens* strains was further confirmed by Dashiff and Kadouri (2011). The strains were co-cultured with *B. bacteriovorus* under optimal conditions, i.e., aerobic atmosphere at 30°C with mild shaking for 48 h. Control samples consisting of the filtered and sterilized lysate formed by BALO predation on *E. coli* were also set up. It was found that the elimination of the analyzed microaerophiles was solely dependent on predator activity, not culture conditions. However, no predation was observed on the model prey strain *E. coli* ZK2686 under microaerophilic or anaerobic conditions, questioning the possibility of using BALOs as an effective therapy against oral anaerobic pathogens. In addition, while *F. nucleatum* PK1594 was found to be susceptible to *B. bacteriovorus* 109 J, other anaerobes such as *F. nucleatum* ATCC10953, *P. intermedia* ATCC 25611, *P. gingivalis* ATCC 33277 and W83, and *T. forsythia* ATCC 43037 were not. The authors did not speculate much on this matter.

*B. bacteriovorus* 109 J application achieved more promising results against *A. actinomycetemcomitans* and *E. corrodens* biofilms (Dashiff and Kadouri, 2011). The predator was able to efficiently eliminate both bacterial biofilms, even in the presence of saliva, which is known protective barrier containing many types of microbial peptides (Abiko and Saitoh, 2007; Gorr, 2009). Interestingly, biofilm removal could be enhanced by applying the DspB enzyme, which can degrade poly-N-acetylglucosamine (PGA, PNAG), a known component of extracellular polymeric substance (EPS) in biofilm. Other enzymes known to target EPS, such as DNase I or proteinase K, did not support the effectiveness of *B. bacteriovorus* 109 J in controlling biofilm (Dashiff and Kadouri, 2011).

A comprehensive study from 2015 analyzed the predation efficacy of *B. bacteriovorus* HD100 against various prey bacteria (Loozen et al., 2015). The first experimental approach consisted of multispecies *in vitro* plate culture, followed by selection on appropriate media after three hours of incubation with a predator, in conditions favorable for the pathogen. The second *ex vivo* approach examined the saliva and subgingival plaque collected from patients with periodontitis using viability qPCR (v-qPCR), i.e., by analysis of the viable cells (Trinh and Lee, 2022) and electrophoresis in a denaturing factor gradient (DGGE) with separation of 16S rRNA coding fragments (Muyzer et al., 1993). The material for v-qPCR and DGGE analyses was collected after 24 h incubation with the predator under aerobic conditions.

In the first approach (Loozen et al., 2015), six periodontitis-associated species were studied: *P. intermedia* ATCC 25611, *A. actinomycetemcomitans* ATCC 43718, *P. gingivalis* ATCC 33277, *F. nucleatum* ATCC 10953, *Streptococcus mitis* ATCC 49456 and *Actinomyces naeslundii* ATCC 52655. The last two species (as Gram-positive bacteria they are not a main target for BALOs (Waso et al., 2021)) served as decoy bacteria as it was previously shown that non-target bacteria may influence predation (Hobley et al., 2006). The second approach included a mixture of four bacterial species: *P. intermedia*, *A. actinomycetemcomitans*, *P. gingivalis*, and *F. nucleatum*.

Both experimental approaches, *in vitro* and *ex vivo*, demonstrated that *B. bacteriovorus* HD100 was able to eliminate *F. nucleatum* and *A. actinomycetemcomitans* without specific preference. The results for *P. intermedia* and *P. gingivalis* were difficult to interpret, as both species declined over time in the control sample without predator. For *P. gingivalis*, negative interactions were proposed between the mixed species particularly with *A. naeslundii*, whereas for *P. intermedia*, the v-qPCR results implied the formation of a viable but nonculturable (VBNC) state, regardless of the presence of *B. bacteriovorus*. In addition, it was observed that the predator promoted the growth of *A. naeslundii* and *S. mitis*: two species associated with both the disease and non-pathogenic oral microbiota. This phenomenon was attributed to an increment in the availability of nutrients as a result of predation on other species (Loozen et al., 2015). In summary, this work further demonstrates the efficacy of BALOs against *F. nucleatum* and *A. actinomycetemcomitans*, but also indicates the complexity of interactions within multispecies systems and the need for further research, including *in vivo* models.

Patini et al. (2019) evaluated the predatory abilities of *B. bacteriovorus* HD100 against several bacterial species, including *E. coli* ATCC 11229, *E. corrodens* ATCC 23834, *F. nucleatum* ATCC 25586, *A. actinomycetemcomitans* ATCC 43717 and *P. gingivalis* ATCC 33277 (Patini et al., 2019). The experiment involved a series of single-species prey–predator co-cultures that were analyzed spectrophotometrically by loss of optical density (OD600). Unlike previous studies, the cultures were carried out for 48 h under aeration and temperature (37°C) conditions suitable for pathogens, rather than predators (30°C). The results indicated that *B. bacteriovorus* is capable of preying on aerobic bacterial species such as *E. coli* and *E. corrodens*, as well as microaerophilic species like *A. actinomycetemcomitans*. However, it did not affect the abundance of anaerobic bacteria like *F. nucleatum* and *P. gingivalis* (Patini et al., 2019). This lack of predation on *F. nucleatum* might be associated with previously-noted strain specificity (Van Essche et al., 2009; Dashiff and Kadouri, 2011; Loozen et al., 2015) but it is more likely due to the fact that the study was performed under prolonged (48 h) anaerobic conditions compared to other studies, which usually use three-hour incubations: *B. bacteriovorus* HD100 has rather low tolerance to reduced oxygen concentrations, which might decrease its capacity for predation (Williams and Piñeiro, 2007).

A different research approach was used by Passariello et al. (2019). The researchers collected samples of oral and dental biofilm from adults of both sexes and extracted metagenomic DNA. The DNA was then used as a template for *B. bacteriovorus*-specific PCR reactions. The study aimed to determine if *B. bacteriovorus* is naturally present in human oral biofilm samples. It was found that all dental biofilm samples, taken from the marginal region of a molar/premolar, and 60% of oral biofilm samples, taken from the upper vestibular mucosa, were positive for *B. bacteriovorus*. Based on the data obtained, the authors suggest that *B. bacteriovorus* plays an important role in balancing the oral microbiota and recommend further research into the use of predatory bacteria for preventing and treating oral diseases (Passariello et al., 2019).

The first *in vivo* studies, conducted in 2019, analyzed the effect of *B. bacteriovorus* HD100 on experimental periodontitis in rats (Silva et al., 2019). The rats were divided into four groups: C (control), EP (experimental periodontitis), C-HD100 and EP-HD100. Experimental periodontitis was induced by ligation of mandibular first molars (MFM) with cotton floss, which resulted in increased colonization of bacteria on the surface of teeth and periodontal tissues (ligature model). In the C-HD100 and EP-HD100 groups, a suspension containing *B. bacteriovorus* HD100 was topically administered on the MFM on days 0, 3, and 7. At the end of the experiment, the animals were sacrificed, and biofilm (formed on ligatures) and gingival tissue were collected for further study. The biofilms were used for qualitative microbiological analysis (DNA hybridization) and quantitative determination of *B. bacteriovorius* (qPCR).

The samples were subjected to immunological analysis to determine the levels of osteoprotegerin, a factor involved in bone remodeling, and the nuclear activator receptor of factor kappa-B (RANKL), which plays a key role in the transcription of genes associated with inflammation. In addition, gene expression was analyzed to determine the production of anti-inflammatory IL-10 and pro-inflammatory IL-17, which also confers antimicrobial effects, as well as FOXP3, the master regulator regulatory T cells function, using qRT-PCR. The samples were also subjected to bone structure imaging using micro-computed tomography and histomorphometric analysis of periodontal tissues (Silva et al., 2019).

It was found that the topical application of predatory bacteria can reduce bone loss and improve bone microstructure, while also protecting affected tissues. The rats treated with *B. bacteriovorus* demonstrated a decrease in the number of bacteria involved in the development of periodontitis, such as *P. intermedia*, *Peptostreptococcus micros*, *F. nucleatum*, *F. polymorphum*, *E. corrodens*, *E. nodatum*, *C. gracilis*, *C. sputigena*, and *V. parvula*-like species. This was accompanied by an increase in the abundance of *Actinomyces viscosus*, *Actinomyces gereneseriae*, and *S. sanguinis*-like species, which are commonly associated with periodontal health (Silva et al., 2019). A similar outcome was observed with *A. naeslundii* and *S. mitis* (Loozen et al., 2015).

Taken together, these findings demonstrate that BALOs have significant potential to be used for the treatment of periodontitis. However, it remains uncertain whether the outcome was a result of *in vivo* predation or an immune response triggered by bacterial components from *B. bacteriovorus*, whether from live or dead cells. Therefore, further research in this area is necessary.

The same research group conducted similar experiments on the ligature periodontitis rat model based on morphometric and immunohistochemical analyses of periodontal tissue, and an expanded enzymatic immunoassay panel (Silva et al., 2023). The immunohistochemical analysis aimed to detect antimicrobial peptides—beta-defensins (BD-1, BD-2, and BD-3) and various markers or receptors associated with immune responses—Toll-Like Receptors (TLR-2, TLR-4)—CD antigens: receptors and ligands (CD-4, CD-8, and CD-57) whereas the enzymatic assays included numerous cytokines that are essential for the proper functioning of the immune system (IL-6, TNF-α, MCP-1, IL-10, IL-1 β, TGF-β, RANTES).

Again, the study confirmed that the topical use of *B. bacteriovorus* HD100 modulated the immune response of periodontal tissues by increasing the immunolabeling pattern for BD-1, BD-2 and BD-3, with higher levels of MCP-1, RANTES, IL-10 and TGF-β, and lower levels of TNF-α compared to untreated periodontitis (Silva et al., 2023). Morphometric analysis indicated that *B. bacteriovorus* had a beneficial influence on alveolar bone loss during periodontitis. Unfortunately, the microbiological composition remains unknown as the analysis was not included in the study.

In summary, the findings demonstrate that treatment with *B. bacteriovorus* HD100 has positive effects on periodontitis. However, the authors note that it remains unclear whether the observed effects were due to predation by bacteria *in vivo* or to the immunoinflammatory host response generated by bacterial components (Silva et al., 2023).

## 6 Future perspective

In periodontal disease which is common and multifactorial both the microbial component and host response play a crucial role. Taking this into account clinical management in periodontitis remained and still remains a staged therapy. Each of the four stages of treatment includes various procedures aimed always at eliminating causative factors, modulating the host’s immune response, and rebuilding the effects of the disease through repair or regenerative procedures.

In recent years we have seen an increasing desire to supplement these approaches with locally- or systemically-administered compounds that can modulate the host response. The aim in such cases is to ameliorate the inflammatory reaction to dysbiosis and foster more favorable clinical parameters of the periodontium compared to mechanotherapy alone or the use of antibiotics or antiseptics. These compounds include statins (reducing inflammation in the periodontal tissues and reducing the level of pro-inflammatory cytokines, stimulating angiogenesis and bone formation pathways) probiotics (altering the conditions in microenvironment niches, interrupting the prevailing dysbiosis, and modulating the immunological-inflammatory response) bisphosphonates (inhibiting osteoclast activity and metalloproteinases, and slowing apoptosis of osteoblasts) polyunsaturated fatty acids Omega-3 (lipid mediators reducing the inflammatory reaction) non-steroidal anti-inflammatory drugs (altering the inflammatory response to the dysbiotic biofilm destroying connective tissue and bone in the periodontium) metformin (activating osteoblasts and inhibiting osteoclasts, reducing oxidative stress and inflammation in the perio-tissues).

Despite the beneficial preliminary clinical effects, all these host-modulating compounds have side effects. Hence, to be considered as the standard treatment recommended by the EFP for the therapy of periodontal diseases, further large multicenter, randomized clinical trials are needed; these should also include multi-level statistical models addressing the occurrence of confounding factors.

At the moment, the future seems to be combining methods aimed at the host (taking into account immune reactions) and at the bacteria themselves.

The best path to success would appear to move towards therapies that consider a greater and more effective reduction of bacterial pathogens, these being the main etiological factors of periodontitis. Hence, phage and bacterio-therapies appear to fulfill this need as they can reduce the bacterial burden with high specificity without damage to host tissues as shown for many diseases of bacterial origin. However, the oral cavity microbiome is very complex and sturdy—structured as a multispecies biofilm and relatively resistant to harsh environmental conditions such as constant flow of saliva or mechanical removal by swallowing food or hygienic treatments. Moreover, many oral pathogens are very difficult to cultivate, therefore, their pathogenesis, as well as predators, are poorly understood or undiscovered. Most of the identified phages aimed at oral bacteria were either temperate (thus not a first choice for therapy) or only partially characterized. The lack of further published investigations indicates that the results obtained were unsatisfactory, leaving phage therapy still an opportunity, but not easily fulfilled.

For the second treatment option—bacteriotherapy, there were more positive results obtained. It was shown that BALOs can kill some oral pathogens during co-culture experiments, mostly aerobes or microaerobes such as *E. corrodens* or *A. actinomycetemcomitans*, respectively. However, experiments were conducted for a short period of time and under aerobic conditions; hence, even when effective against anaerobes (*F. nucleatum*), it is hard to estimate if it would be as effective in dynamic (oral cavity) and anaerobic environment (within biofilm). Few *in vivo* experiments showed positive treatment effects with *B. bacteriovorus* HD100 on experimental periodontitis in rats. Although the exact mechanisms were not specified, it was proposed that BALOs induced beneficial immunological events that led to the recovery of the affected tissue (bone). Thus, bacteriotherapy remains a promising therapy that needs further research.

Of course, other alternative approaches have been investigated, such as vaccination, non-antibiotic treatments such as silver and antimicrobial peptides (solo or encapsulated in liposomes), as well as phototherapy, plant compounds such as garlic, vitamins, enzymatic antioxidants or ozone treatment. Future studies could also consider variations of EFP, such as molecular modification of phages, the use of endolysins instead of whole phages and combinatorial therapy: this can involve combinations of phages and predatory bacteria, two kinds of phages with antibiotics, or the precise delivery of antibiotics using liposomes in order to first penetrate or destroy biofilm and then kill pathogens.

## 7 Concluding remarks

Periodontal disease is not only caused by a variety of factors but also tends to progress in a difficult manner, often leading to various complications and affecting a large number of people. It should be emphasized that the treatment is complex, requiring many phases of therapy, and difficult to implement successfully due to bacterial resistance and allergic reactions. Moreover, it is associated with high costs of treatment and local and systemic complications. There is therefore a pressing need for innovative research to identify new agents and treatment methods. One such approach involves the development and standardization of therapy using bacteriophages or predatory bacteria, which have been successfully used in the fight against pathogens in other diseases.
